# FaXNet: a frequency-adaptive, explainable, and uncertainty-aware network for influenza forecasting

**DOI:** 10.3389/fpubh.2026.1746529

**Published:** 2026-02-02

**Authors:** Wei He, Xuanfeng Li, Xiaolin Liang, Zige Liu, Zhiqi Zeng, Zifeng Yang, Chitin Hon

**Affiliations:** 1 Faculty of Innovation Engineering, Macau University of Science and Technology, Macao, Macao SAR, China; 2 Institute of Systems Engineering, Macau University of Science and Technology, Macao, Macao SAR, China; 3 Faculty of Social Sciences, The University of Hong Kong, Hong Kong, Hong Kong SAR, China; 4 National Clinical Research Center for Respiratory Disease, State Key Laboratory of Respiratory Disease, Guangzhou Institute of Respiratory Health, The First Affliated Hospital of Guangzhou Medical University, Guangzhou, Guangdong, China

**Keywords:** Adaptive Fourier Decomposition (AFD), explainable AI (XAI), frequency-adaptive modeling, influenza forecasting, Long Short-Term Memory (LSTM), SHAP (SHapley Additive exPlanations)

## Abstract

**Background:**

Accurate and interpretable influenza forecasting is critical for public health preparedness, yet many models struggle to capture multi-scale temporal dynamics and to provide reliable uncertainty estimates. These challenges are particularly pronounced in China, where influenza seasonality differs between northern temperate and southern subtropical regions.

**Methods:**

We propose FaXNet, a frequency-adaptive, explainable, and uncertainty-aware deep learning framework that integrates data-driven spectral representation with interpretable component selection and probabilistic forecasting. We compiled weekly influenza positivity rates from the Chinese National Influenza Center and aligned them with ERA5-Land meteorological variables (temperature, dew point, and precipitation) for northern and southern China from 2011 to 2023. FaXNet was evaluated against representative statistical, machine learning, deep learning, and decomposition-based baselines for 1–4-week-ahead forecasting using standard accuracy and calibration metrics.

**Results:**

FaXNet achieved consistently superior performance in both regions, with 1-week-ahead R^2^ of 0.9319 (north) and 0.8665 (south), and 4-week-ahead R^2^ of 0.4493 (north) and 0.4960 (south). The proposed method maintained a statistically significant performance advantage against all benchmarks across varying horizons, validating the effectiveness of frequency-adaptive modeling in mitigating error accumulation. Model explanations highlighted precipitation as the dominant meteorological driver in the north and temperature as the primary factor in the south.

**Conclusion:**

FaXNet provides accurate, interpretable forecasts with calibrated prediction intervals across 1–4-week horizons, offering actionable lead time for region-specific risk assessment and resource planning. Performance may depend on surveillance data completeness and the limited set of exogenous covariates considered, motivating future extensions with additional drivers (e.g., mobility or vaccination) and broader external validation.

## 1 Introduction

Influenza continues to pose a major global health threat, leading to millions of infections annually and maintaining the potential for pandemic outbreaks (1, 2). The rapid antigenic drift of influenza viruses and the possibility of zoonotic transmission underscore the importance of accurate and timely forecasting for preparedness and response (3, 4). Reliable forecasts enable health authorities to allocate medical resources, implement targeted interventions, and communicate risks effectively (5, 6). At the same time, influenza transmission reflects interactions among seasonal cycles, meteorological drivers, and human behavior that unfold across multiple temporal scales (7, 8). Forecasting systems must therefore represent these multi-scale dynamics while offering interpretability and uncertainty quantification to inform public health decision-making (9, 10).

Substantial evidence links meteorological conditions to influenza activity (11, 12). Temperature, humidity, and precipitation influence viral survival, host susceptibility, and social mixing, with effects that vary in magnitude and direction across regions and seasons. In China, the climatic contrast between the northern temperate and southern subtropical provinces creates distinct epidemic profiles, motivating region-specific models that can adapt to environmental heterogeneity (13). Because these drivers operate over time scales ranging from weekly fluctuations to annual cycles, frequency-dependent representations can more faithfully capture their effects in epidemiological time series (14, 15). Crucially, epidemiological series are inherently non-stationary, characterized by time-varying amplitudes and frequencies that traditional fixed-basis methods often fail to resolve.

Recent advances in time-series forecasting (TSF) aim to address non-stationarity and multi-scale temporal structures through both representation learning and frequency-aware modeling. TimesNet (16), for example, exploits multi-periodicity by reorganizing one-dimensional sequences into two-dimensional representations to learn heterogeneous temporal patterns, while FEDformer (17) integrates seasonal–trend decomposition with frequency-domain enhancement to better capture long-range dynamics. More recently, inverted transformer architectures have further demonstrated the efficacy of capturing multivariate correlations and temporal dependencies explicitly (18). In epidemiological settings—where interpretability and periodic structure are central—spectral analysis yields actionable insights into epidemic cycles and structural changes, as demonstrated in recent studies of COVID-19 surveillance dynamics (19). At the population level, large-scale collaborative influenza forecasting efforts further underscore that transparency and interpretability are essential when model outputs inform critical public health decision-making (20, 21). Despite these advances, many state-of-the-art TSF models remain difficult to interpret at a granular, frequency-specific level, and the systematic integration of spectral decomposition with principled game-theoretic attribution frameworks, such as SHAP (22), remains limited in operational forecasting pipelines.

In the specific context of influenza-related public health modeling, these challenges are further compounded by several persistent limitations (9). First, feature selection is often *ad hoc* or conducted *post hoc*, hindering the principled identification of the most relevant temporal scales and frequency components, which in turn limits interpretability (23, 24). Second, while classical spectral techniques effectively encode seasonality, they are rarely integrated with modern, interpretable selection mechanisms, constraining their explanatory value in real-world operational pipelines (19, 25). Third, many studies continue to emphasize point forecasts without rigorous uncertainty quantification, reducing their usefulness for risk-sensitive planning and evaluation (20). Collectively, these unresolved issues impede the development of transparent, uncertainty-aware tools suitable for routine clinical and public health deployment.

To address these gaps, we propose FaXNet (Frequency-Adaptive eXplainable Network), a forecasting framework that integrates Adaptive Fourier Decomposition (AFD), SHapley Additive exPlanations (SHAP)-based feature selection, and a Frequency-Adaptive Long Short-Term Memory (FALSTM) model with probabilistic deep learning. AFD derives data-driven frequency components to capture multi-scale influenza–meteorology structures, addressing the limitations of fixed-basis transforms such as the Fast Fourier Transform (FFT) (26, 27). We then apply a SHAP-based selection strategy to identify the most predictive components and quantify their marginal contributions within a game-theoretic attribution framework (28, 29). The selected components are processed by the FALSTM with spectral fusion and an exogenous gate, enabling adaptive weighting of frequency components conditional on meteorological covariates (30). A probabilistic decoder produces point forecasts together with calibrated uncertainty estimates, which are evaluated using coverage-based metrics similar to those employed in FluSight frameworks, thereby supporting risk-aware public health decisions (31).

Contributions and novelty. To address these challenges, we propose FaXNet (Frequency-Adaptive eXplainable Network), a framework that integrates adaptive frequency representation, explainable component selection, and probabilistic multi-horizon forecasting. Our contributions are fourfold: (i) a unified end-to-end design that jointly captures multi-scale dynamics, interpretability, and uncertainty for influenza prediction; (ii) the use of AFD to obtain data-driven frequency components from epidemiological and meteorological series as interpretable learning channels; (iii) a SHAP-guided selection strategy that yields compact inputs and transparent attribution of influential temporal scales and meteorological drivers; and (iv) probabilistic 1–4-week-ahead forecasting with prediction intervals, validated across northern and southern China (2011–2023) against representative baseline families.

Paper organization. Section 2 introduces the datasets, preprocessing, and the FaXNet methodology. Section 3 reports benchmark comparisons, ablation studies, multi-horizon results, and interpretability and uncertainty analyses. Section 4 discusses implications, limitations, and future directions. Section 5 concludes the paper.

## 2 Materials and methods

This section presents the FaXNet framework for influenza forecasting, which integrates adaptive spectral decomposition, interpretable feature selection, and probabilistic neural architecture to effectively leverage meteorological covariates.

### 2.1 Problem formulation

Let $D={\left\{(y_{t},e_{t})\right\}}_{t=1}^{T}$ denote the dataset, where $y_{t}\in \mathbb{R}$ represents the influenza positivity rate at time t, and $e_{t}\in {\mathbb{R}}^{J}$ contains J meteorological features. For a historical window of length L and forecasting horizon H, the input–output mapping is:


$$X_{s}=(y_{s},\ldots ,y_{s+L-1}),y_{s}=(y_{s+L},\ldots ,y_{s+L+H-1})$$

The data is chronologically split into training (70%), validation (10%), and test (20%) sets. Exogenous features are standardized using z-score normalization: ${\tilde{e}}_{t,j}=(e_{t,j}-\mu_{j})/\sigma_{j}$, where $\mu_{j}$ and $\sigma_{j}$ are the mean and standard deviation of feature j.

### 2.2 Dataset

We construct two regional weekly datasets by integrating influenza surveillance from the Chinese National Influenza Center (CNIC) with land-surface meteorology from ERA5-Land. The datasets cover the period from January 1, 2011, to December 17, 2023, encompassing over 12 years of continuous weekly observations. The targets are the weekly influenza positivity rates for Northern China and Southern China, indexed by CNIC’s reporting weeks. CNIC defines these two regions following standard epidemiological boundaries used in China’s influenza surveillance system.

Meteorological covariates are drawn from ERA5-Land hourly single-level fields: 2 m air temperature (t2m), 2 m dew-point temperature (d2m), and total precipitation (tp). We define the northern and southern masks based on provincial membership (for example: provinces located north of ~33° N latitude are assigned to Northern China, the others to Southern China) (27). For each region, we average the values of all land grid cells within the mask to produce a regional hourly series. We then temporally aggregate hourly data into CNIC-aligned weekly time steps using simple weekly means for t2m, d2m, and tp, producing synchronized covariate series aligned with influenza positivity rates.

### 2.3 Adaptive Fourier decomposition (AFD)

Discrete inner product and analytic boundary. Let $t_{n}=\frac{2\mathrm{\pi n}}{T}$, $z_{n}=e^{{\mathrm{it}}_{n}}\in T$. We discretize the Hardy inner product as (27):


$${\langle f,g\rangle }_{w}=\sum_{n=1}^{T}w_{n}f(z_{n})\bar{g(z_{n})},w_{n}\geq 0,\sum_{n}w_{n}=1$$ (1)

and construct the analytic extension of the real series by suppressing negative frequencies (Hilbert transform), using boundary samples $f(z_{n})$.

Higher-order Szegő atoms and residual update. For pole a∈D={z:∣z∣<1} and order $m\in {\mathbb{N}}_{0}$:


$$B_{a}^{\left(m\right)}(z)=\frac{m!}{{\left(1-\bar{a}z\right)}^{m+1}}z^{m}$$ (2)

Initialize $c_{0}={\langle f,1\rangle }_{w}$, $f_{0}=f$. At step k, select $a_{k}$ on a finite grid C⊂D by normalized maximal selection (27):


$$\begin{array}{c} a_{k}=\arg\max_{a\in C}\frac{{|{\langle f_{k-1},B_{a}^{\left(m\right)}\rangle }_{w}|}^{2}}{{\langle B_{a}^{\left(m\right)},B_{a}^{\left(m\right)}\rangle }_{w}},c_{k}=\frac{{\langle f_{k-1},B_{a_{k}}^{\left(m\right)}\rangle }_{w}}{{|B_{a_{k}}^{\left(m\right)}|}_{2,w}^{2}} \end{array}$$ (3)

and update the residual via the Möbius transform:


$$\begin{array}{c} M_{a}(z)=\frac{z-a}{1-\bar{a}z},f_{k}(z)=\frac{f_{k-1}(z)-c_{k}B_{a_{k}}^{\left(m\right)}(z)}{M_{a_{k}}(z)} \end{array}$$ (4)

Reconstruction, primitive channels, and energies. The K-term AFD reconstruction is:


$$\begin{array}{c} {\hat{f}}_{K}(z)=c_{0}+\sum_{k=1}^{K}c_{k}B_{a_{k}}^{\left(m\right)}(z)\prod_{j=1}^{k-1}M_{a_{j}}(z) \end{array}$$ (5)

For learning, we deliberately use primitive atom contributions (approximation):


$$\begin{array}{c} c_{n}^{\left(k\right)}=ℜ[c_{k}B_{a_{k}}^{\left(m\right)}(z_{n})],n=1,\ldots ,T, \end{array}$$ (6)

which yield one interpretable channel per atom, enabling parallel encoders. We define global and window energies:


$$\begin{array}{c} \begin{array}{l} E_{\text{full}}^{\left(k\right)}=\frac{1}{T}\sum_{n=1}^{T}{\left(c_{n}^{\left(k\right)}\right)}^{2},E_{\mathrm{win},s}^{\left(k\right)}=\frac{1}{L}\sum_{\tau =1}^{L}{\left(x_{s,\tau }^{\left(k\right)}\right)}^{2},\\ x_{s}^{\left(k\right)}=(c_{s}^{\left(k\right)},\ldots ,c_{s+L-1}^{\left(k\right)}) \end{array} \end{array}$$ (7)

Stopping rule. With ${|g|}_{2,w}=\sqrt{\sum_{n}\;\;w_{n}{|g(z_{n})|}^{2}}$, stop when:


$$\begin{array}{c} \frac{{|ℜ(f)-ℜ({\hat{f}}_{K})|}_{2,w}}{{|ℜ(f)|}_{2,w}+\delta }\leq \varepsilon \;\text{or}\;K=K_{\max},\delta >0 \end{array}$$ (8)

### 2.4 SHAP-guided component selection

Stack all target- and exogenous-derived atoms into $C\in {\mathbb{R}}^{K_{\mathrm{all}}\times T}$. For windows:


$$\begin{array}{c} F_{s,k}=c_{s+L-1}^{\left(k\right)},F\in {\mathbb{R}}^{N\times K_{\mathrm{all}}},y_{s}^{\left(h\right)}=y_{s+L-1+h},h=1,\ldots ,H. \end{array}$$ (9)

Fit a random-forest surrogate $\hat{f}$ on training windows (multi-output across h or per-h models). TreeExplainer gives Shapley values (20):


$$\begin{array}{c} \begin{array}{l} \phi_{s,k}=\sum_{S\subseteq K\backslash \{k\}}\frac{|S|!(K-|S|-1)!}{K!}[\hat{f}(F_{s,S\cup \{k\}})-\hat{f}(F_{s,S})],\\ K=\{1,\ldots ,K_{\mathrm{all}}\} \end{array} \end{array}$$ (10)

Global importance $I_{k}=\frac{1}{N}\sum_{s=1}^{N}|\phi_{s,k}|$. Select $K^{*}$ as Top-$K^{*}$ by $I_{k}$ with an energy-coverage constraint:


$$\begin{array}{c} \sum_{k\in K^{*}}E_{\text{full}}^{\left(k\right)}\geq \eta \sum_{k=1}^{K_{\mathrm{all}}}E_{\text{full}}^{\left(k\right)},\eta \in (0,1) \end{array}$$ (11)

supplementing target-derived atoms if needed. Optionally, append the raw target window $X_{s}$ as a time-domain channel.

### 2.5 Frequency-aware LSTM (FALSTM)

Encoders. For each $k\in K^{*}$, an LSTM encodes $x_{s}^{\left(k\right)}\in {\mathbb{R}}^{L}$ into the final hidden state (30) $h^{\left(k\right)}\in {\mathbb{R}}^{d}$; we also carry $E_{\mathrm{win},s}^{\left(k\right)}\in \mathbb{R}$:


$$\begin{array}{c} x_{s}^{\left(k\right)}\overset{\;\text{LSTM}\;\;}{\to }h^{\left(k\right)}\in {\mathbb{R}}^{d},E_{\mathrm{win},s}^{\left(k\right)}\in \mathbb{R} \end{array}$$ (12)

Spectral gating and fusion. Compute a spectral logit via an MLP:


$$\begin{array}{c} \omega_{k}=v^{⊤}\psi (W_{h}h^{\left(k\right)}+w_{E}E_{\mathrm{win},s}^{\left(k\right)}+b),\omega_{k}\in \mathbb{R} \end{array}$$ (13)

and softmax weights (temperature τ>0; numerically stabilized by subtracting $\max_{j}\omega_{j}$ in practice):


$$\begin{array}{c} \alpha_{k}=\frac{\exp(\omega_{k}/\tau )}{\sum_{j\in K^{*}}\exp(\omega_{j}/\tau )},\alpha \in \Delta^{K^{*}-1} \end{array}$$ (14)

then form the fused state:


$$\begin{array}{c} h_{f}=\sum_{k\in K^{*}}\alpha_{k}h^{\left(k\right)}\in {\mathbb{R}}^{d} \end{array}$$ (15)

Exogenous gating. Summarize exogenous inputs by mean and last value:


$$\begin{array}{c} z_{s}=[\text{mean}({\tilde{E}}_{s:s+L-1}),{\tilde{e}}_{s+L-1}]\in {\mathbb{R}}^{2J} \end{array}$$ (16)

and apply a sigmoid gate (23):


$$\begin{array}{c} g=\sigma (W_{g}z_{s}+b_{g})\in {\left(0,1\right)}^{d},W_{g}\in {\mathbb{R}}^{d\times 2J},\tilde{h}=g⊙h_{f}\in {\mathbb{R}}^{d} \end{array}$$ (17)

Probabilistic decoder. With layer normalization and linear heads:


$$\begin{array}{c} \mu =W_{\mu }\phi (\mathrm{LN}(\tilde{h}))+b_{\mu },\log\sigma^{2}=W_{\sigma }\phi (\mathrm{LN}(\tilde{h}))+b_{\sigma } \end{array}$$ (18)

clamping $\log\sigma^{2}\in [a,b]$ (e.g., [−10,10]); set $\sigma^{2}=\exp(\log\sigma^{2}).$ The predictive distribution is:


$$\begin{array}{c} p(y_{s}|X_{s})=N(\mu ,\mathrm{diag}(\sigma^{2})) \end{array}$$ (19)

### 2.6 Learning objective and optimization

Gaussian NLL with MSE regularization (9), as defined in Equations 20 and 21:


$$\begin{array}{c} L_{\mathrm{NLL}}=\frac{1}{\mathrm{NH}}\sum_{s=1}^{N}\sum_{h=1}^{H}\frac{1}{2}[\log(2\pi )+\log\sigma_{s,h}^{2}+\frac{{\left(y_{s}^{\left(h\right)}-\mu_{s,h}\right)}^{2}}{\sigma_{s,h}^{2}}] \end{array}$$ (20)

$$\begin{array}{c} L=L_{\mathrm{NLL}}+\lambda \cdot \frac{1}{\mathrm{NH}}\sum_{s,h}{\left(y_{s}^{\left(h\right)}-\mu_{s,h}\right)}^{2},\lambda \geq 0 \end{array}$$ (21)

We use AdamW (lr = 1 × 10^−3^), mini-batches, gradient-norm clipping, and early stopping (patience = 15); hyperparameters were tuned via rolling-origin grid search with lr ∈ {1 × 10^−4^, 5 × 10^−4^, 1 × 10^−3^}, LSTM hidden size h ∈ {64, 128, 256} (selected 128), dropout p ∈ {0, 0.1, 0.2} (selected 0.1), AFD atoms K ∈ {6, 8, 10} (selected 8), and SHAP-selected features m ∈ {8, 10, 12, 14} (selected 12).

### 2.7 Evaluation and uncertainty diagnostics

Point forecasts are evaluated using the metrics defined in Equations 22 and 23:


$$\begin{array}{c} \begin{array}{l} \mathrm{MSE}=\frac{1}{\mathrm{NH}}\sum_{s,h}{\left(\mu_{s,h}-y_{s}^{\left(h\right)}\right)}^{2},\text{RMSE}=\sqrt{\mathrm{MSE}},\\ \mathrm{MAE}=\frac{1}{\mathrm{NH}}\sum_{s,h}|\mu_{s,h}-y_{s}^{\left(h\right)}| \end{array} \end{array}$$ (22)

$$\begin{array}{c} R^{2}=1-\frac{\sum_{s,h}{\left(\mu_{s,h}-y_{s}^{\left(h\right)}\right)}^{2}}{\sum_{s,h}{\left(y_{s}^{\left(h\right)}-\bar{y}\right)}^{2}}\;(\bar{y}\;\text{computed}\;\mathrm{on}\;\text{test}\;\mathrm{set}) \end{array}$$ (23)

**Figure 1:**
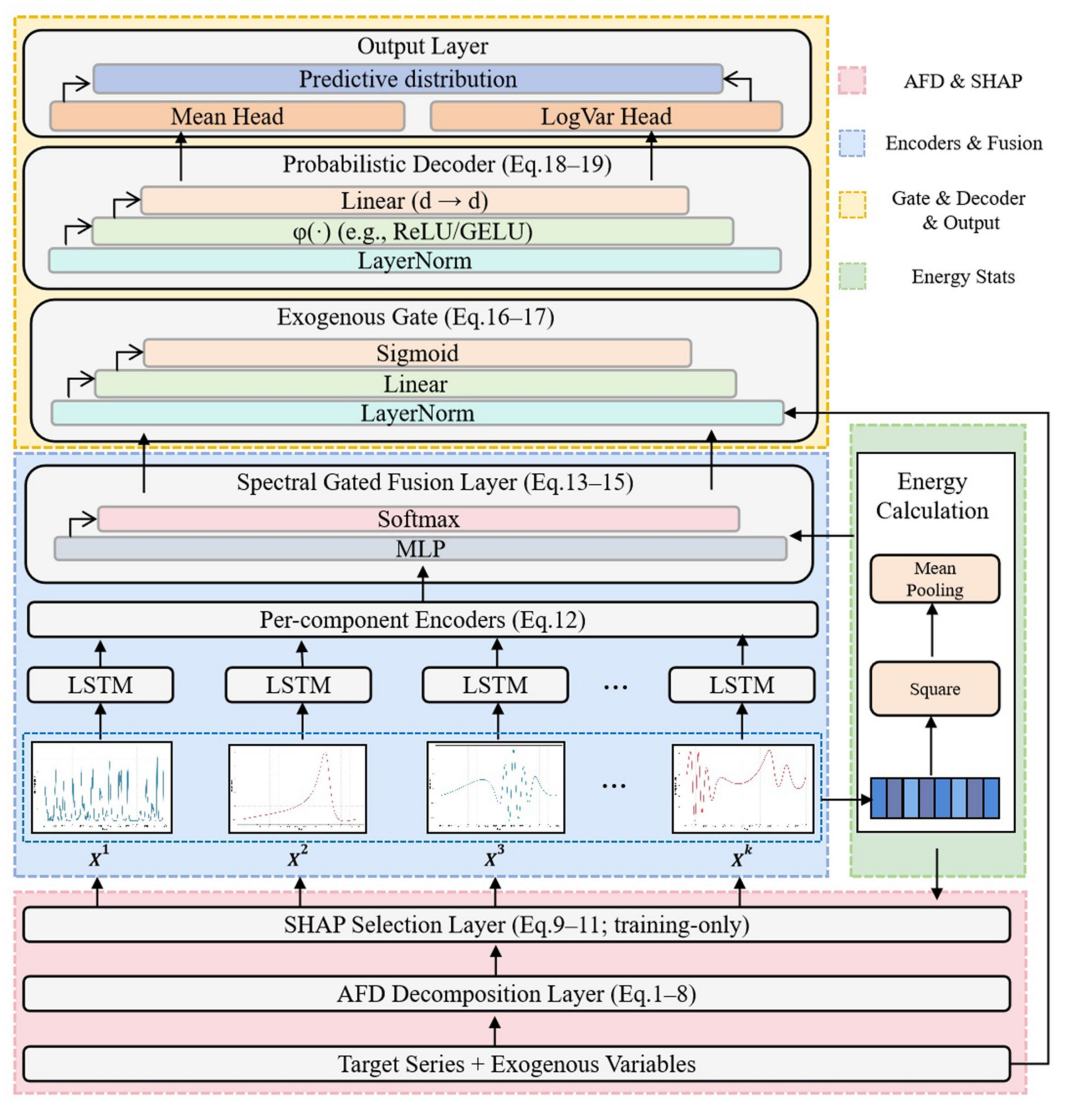
Architecture of the proposed FaXNet framework. Schematic representation of the proposed FaXNet (Frequency-Adaptive eXplainable Network) for influenza forecasting. The framework integrates adaptive Fourier decomposition (Equations 1–8), SHAP-guided feature selection (Equations 9–11), frequency-aware LSTM encoding and spectral gated fusion (Equations 12–15), exogenous gating (Equations 16, 17), and probabilistic decoding (Equations 18, 19). Energy calculation supports spectral weighting. Together, these components enable interpretable, uncertainty-aware, and region-generalizable influenza forecasting.

We additionally report horizon-wise metrics and calibration diagnostics (empirical coverage of (1−α) intervals, PIT/CRPS) (Figure 1).

### 2.8 Experimental design

The experimental evaluation uses influenza surveillance datasets from both northern and southern China to assess the regional generalizability of FaXNet (Frequency-Adaptive eXplainable Network). Weekly influenza positivity rates were obtained from the Chinese National Influenza Center (CNIC) and paired with region-specific meteorological covariates from ERA5-Land reanalysis, covering multiple years at weekly resolution. Consistent preprocessing was applied, including Z-score normalization, and a strictly chronological 70%/10%/20% split (train/validation/test) to prevent temporal leakage; importantly, the test set is a continuous held-out tail segment spanning multiple influenza seasons to reflect deployment-realistic generalization, while the validation set is used exclusively for early stopping and hyperparameter selection. Beyond this single split, we additionally adopt a rolling-origin (expanding-window) protocol on the pre-test timeline, advancing multiple forecast origins and summarizing performance across origins (mean ± std) to strengthen robustness. The model configuration is kept identical across regions (eight AFD components, twelve SHAP-selected features, and a three-layer LSTM with 128 hidden units), and training employs AdamW with a learning rate of 1 × $10^{-3}$, batch size of 32, gradient-norm clipping, and early stopping (patience = 15).

The evaluation protocol comprises four complementary analyses: (i) regional comparative experiments to characterize climate-specific transmission patterns; (ii) comprehensive ablation studies to assess the contribution of each architectural component; (iii) multi-horizon forecasting analysis (one- to four-week ahead) to evaluate short- and medium-term predictive robustness; and (iv) interpretability analysis based on SHAP feature importance to identify region-specific meteorological drivers. This dual-region experimental framework ensures a rigorous and balanced validation of FaXNet across diverse climatic and epidemiological contexts.

Implementation Notes: Under standard assumptions, Adaptive Fourier Decomposition (AFD) with maximal selection converges in the Hardy norm, and Equation 1 provides a consistent quadrature surrogate. Normalization in Equation 3 mitigates near-boundary pole bias arising from the variation of $|B_{a}^{\left(m\right)}|$. Primitive channels in Equation 6 are introduced to enhance interpretability and parallel computation, while Equation 5 governs reconstruction accuracy. The simplex-constrained spectral weights α and exogenous gate $g\in {\left(0,1\right)}^{d}$ bound the fused representation $\tilde{h}$, ensuring numerical stability during optimization.

## 3 Results and discussion

This section reports a comprehensive set of experiments evaluating the proposed FaXNet framework on influenza datasets from both northern and southern China. The analyses aim to demonstrate the effectiveness and generalizability of our approach across distinct climatic conditions. We conduct systematic comparisons against closely related and competitive baselines, assess performance across multiple forecasting horizons, perform rigorous ablation studies to quantify the contributions of individual components, and apply interpretability analyses to reveal region-specific transmission patterns. Together, these experiments provide a holistic validation of our framework from accuracy, robustness, and interpretability perspective.

### 3.1 Comprehensive performance analysis: baseline comparison and multi-horizon prediction

To establish the overall effectiveness of FaXNet, we first integrate baseline method comparisons with multi-horizon forecasting results. This design allows us to evaluate both methodological competitiveness and practical forecasting utility at varying temporal resolutions. Importantly, we report not only pointwise accuracy improvements but also their implications for operational forecasting across horizons and regions, thereby combining results with interpretation in a unified analysis.

#### 3.1.1 Baseline methods and experimental setup

**Figure 2:**
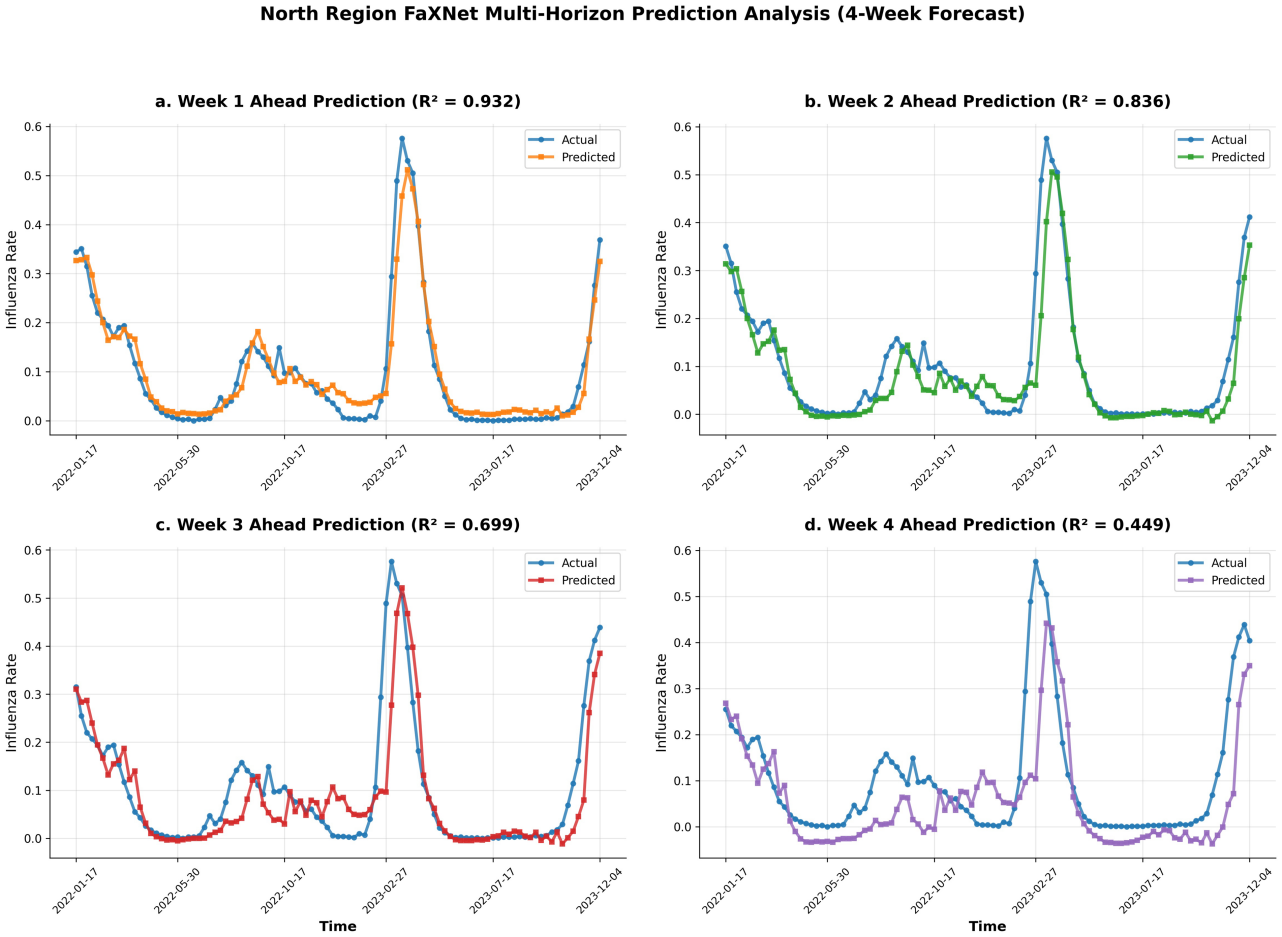
FaXNet multi-horizon influenza forecasts for Northern China. Actual vs. predicted positivity rates across 1–4 week horizons, showing high short-term accuracy and stable medium-range performance. **(a)** Week 1 ahead prediction (R^2^ = 0.932). **(b)** Week 2 ahead prediction (R^2^ = 0.836). **(c)** Week 3 ahead prediction (R^2^ = 0.699). **(d)** Week 4 ahead prediction (R^2^ = 0.449).

We benchmark FaXNet against ten representative baselines spanning diverse methodological families: ARIMA (Autoregressive Integrated Moving Average, statistical), SVR-RBF (Support Vector Regression with Radial Basis Function kernel, machine learning), LSTM (Long Short-Term Memory), GRU (Gated Recurrent Unit), Transformer, TimesNet, and FEDformer, as well as three hybrid decomposition methods: EMD-LSTM (Empirical Mode Decomposition (32) with LSTM), VMD-LSTM (Variational Mode Decomposition (33) with LSTM), and Wavelet-LSTM. Input configurations follow standard practice: statistical and decomposition-based methods (ARIMA, EMD-LSTM, VMD-LSTM, Wavelet-LSTM) are trained exclusively on historical influenza data, whereas machine learning and deep learning methods (SVR-RBF, LSTM, GRU, Transformer) incorporate both influenza and meteorological covariates (temperature, humidity/dewpoint, precipitation). The proposed model further extends this multi-source input with adaptive frequency decomposition and SHAP-guided feature selection to enhance representation learning. This setup enables a fair comparison between univariate/decomposition-based forecasters and multivariate learners leveraging meteorological context, and helps assess the incremental contribution of FaXNet’s frequency-adaptive and explainable design (Figure 2).

All models were trained and evaluated under an identical protocol with a strict chronological 70/10/20 split (train/validation/test). The validation subset was used only for early stopping and hyperparameter selection, while the test subset remained untouched and was evaluated once for final reporting. To prevent look-ahead bias from random k-fold procedures, hyperparameters were tuned by grid search using rolling-origin (expanding-window) evaluation on the pre-test timeline (K = 5 forecast origins): at each origin, models were fit on all available past data and assessed on the subsequent validation block; the configuration minimizing validation MAE (primary) and RMSE/NLL (secondary) was selected, then refit on train+validation before the single test evaluation. We report RMSE, MAE, R^2^, and MAPE, with MAE as the primary ranking metric. Table 1 summarizes average baseline performance (4-week window) across regions and the 1–4 week multi-horizon forecasts of FaXNet. Finally, to rigorously assess statistical significance, we applied a block-wise paired Wilcoxon signed-rank test to the out-of-sample errors on the untouched test set, aggregating predictions into non-overlapping blocks to mitigate temporal autocorrelation.

**Table 1:** Comprehensive performance analysis: baseline comparison and multi-horizon prediction.

Section	Type	Method/Horizon	RMSE	MAE	$R^{2}$	MAPE	RMSE	MAE	$R^{2}$	MAPE
			Northern China				Southern China			
Baseline (4-week avg)	Statistical	ARIMA	0.0965	0.0718	0.472	14.28%	0.1332	0.0957	0.365	18.05%
	ML	SVR-RBF	0.0898	0.0654	0.518	12.95%	0.124	0.0886	0.41	16.62%
	Deep Learning	Transformer	0.0827	0.0596	0.603	11.24%	0.116	0.0817	0.475	14.85%
	Deep Learning	LSTM	0.0785	0.0551	0.661	10.48%	0.1122	0.078	0.502	13.98%
	Deep Learning	GRU	0.0762	0.0536	0.675	10.05%	0.1085	0.0745	0.526	13.42%
	Deep Learning	TimesNet	0.0815	0.0582	0.615	11.10%	0.1158	0.0812	0.482	14.55%
	Deep Learning	FEDformer	0.0732	0.0495	0.685	9.25%	0.1045	0.0702	0.555	12.65%
	Hybrid	EMD-LSTM	0.0741	0.052	0.688	9.71%	0.106	0.0715	0.548	12.89%
	Hybrid	Wavelet-LSTM	0.0728	0.0506	0.697	9.54%	0.1039	0.0695	0.559	12.55%
	Hybrid	VMD-LSTM	0.0719	0.0499	0.705	9.36%	0.1028	0.0682	0.57	12.23%
	Proposed	FaXNet	**0.0698**	**0.0411**	**0.7239**	**7.65%**	**0.0889**	**0.0616**	**0.6778**	**10.12%**
Multi-horizon (FaXNet)	Temporal	1-week forecast	0.034	0.0233	0.9319	4.08%	0.055	0.0379	0.8665	5.68%
	Temporal	2-week forecast	0.0534	0.031	0.8358	5.25%	0.0817	0.0585	0.7194	8.12%
	Temporal	3-week forecast	0.0734	0.0423	0.6994	8.12%	0.1142	0.0715	0.6293	11.89%
	Temporal	4-week forecast	0.1006	0.0678	0.4493	11.25%	0.1156	0.0785	0.496	12.15%

Bold values indicate the best performance for each metric within the corresponding region.

Across both regions and all metrics, FaXNet achieves the strongest overall performance. Compared with ARIMA, it reduces MAE by 40.2% in northern China and 33.3% in southern China, while also attaining the highest R^2^ values (0.7239 in the north and 0.6778 in the south). The framework further demonstrates robust short- to medium-term accuracy, achieving R^2^ > 0.92 at the 1-week horizon and degrading smoothly over longer horizons, remaining practically useful with R^2^ > 0.4 at 4 weeks in both regions. From an interpretive perspective, these improvements are consistent with the hypothesis that combining frequency-adaptive decomposition with attribution-guided selection helps emphasize informative temporal components and mitigate the influence of less relevant variability, an effect that becomes increasingly important for longer-horizon prediction. Notably, performance is consistently stronger in northern China than in southern China. This regional gap may reflect the clearer and more regular seasonality in temperate climates, whereas subtropical dynamics could be more sensitive to higher climatic variability and unmodeled drivers (e.g., mobility, school terms, and strain composition) (34), which may limit achievable accuracy under the current covariate set. Nevertheless, FaXNet maintains a stable advantage over all statistical, machine learning, deep learning, and hybrid baselines across both regions, supporting its robustness and generalizability. In practical terms, the sustained advantage at 3–4 weeks may inform earlier preparedness planning (e.g., resource planning) beyond short-term nowcasting, while the gradual degradation across horizons suggests stable error growth rather than abrupt failure.

**Figure 3:**
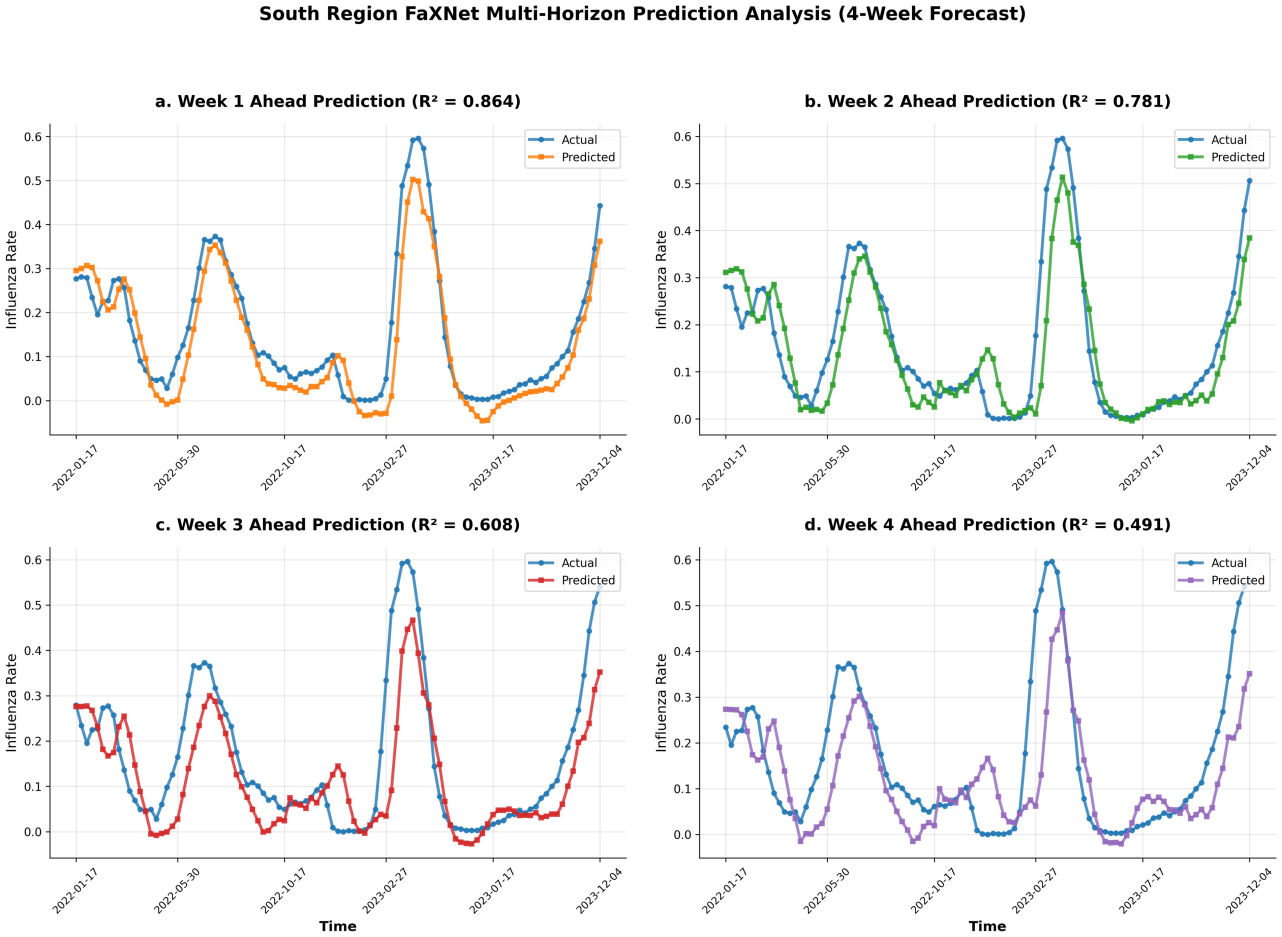
FaXNet multi-horizon influenza forecasts for Southern China. Actual vs. predicted positivity rates across 1–4 week horizons, demonstrating strong short-term accuracy and consistent medium-range performance. **(a)** Week 1 ahead prediction (R^2^ = 0.864). **(b)** Week 2 ahead prediction (R^2^ = 0.781). **(c)** Week 3 ahead prediction (R^2^ = 0.608). **(d)** Week 4 ahead prediction (R^2^ = 0.491).

To rigorously substantiate the model’s robustness beyond point accuracy, we evaluated both its probabilistic calibration and statistical significance. In terms of uncertainty quantification, FaXNet achieved low Continuous Ranked Probability Scores (CRPS) of 0.0285 (North) and 0.0412 (South), with 95% prediction interval coverage rates of 94.2 and 93.8%, respectively. These metrics, widely adopted in epidemic forecasting evaluations (35, 36), confirm that the predictive distributions are well-calibrated and not overly dispersive. Concurrently, to validate that the performance gains were not due to random chance, we applied a block-wise paired Wilcoxon signed-rank test to the rolling-origin forecast errors between FaXNet and the strongest baseline. Results confirmed statistically significant improvements in both Northern and Southern China (*p* < 0.05). Together, these diagnostics demonstrate that FaXNet provides a systematic, scientifically significant, and reliable predictive advantage across distinct climatic regimes (Figure 3).

### 3.2 Ablation study results

We assess the marginal contribution of each component in the proposed framework through a controlled ablation. Starting from the frequency-adaptive LSTM backbone (FALSTM), we progressively introduce Adaptive Fourier Decomposition (AFD) and SHAP-guided feature selection, while keeping the network architecture, training budget, and evaluation protocol consistent across variants to enable a fair component-wise comparison. Metrics are computed on the test set and reported as 4-week averages.

We evaluate four model variants under an identical experimental protocol. The first variant, FALSTM (baseline), represents the base frequency-adaptive LSTM that incorporates spectral fusion and exogenous gating, trained directly on raw time series without applying decomposition or feature selection. The second variant, AFD-FALSTM, extends the baseline by integrating adaptive frequency decomposition, while the third variant, SHAP-FALSTM, augments the baseline with SHAP-based feature selection. The final variant, FaXNet (proposed), combines both adaptive decomposition and SHAP-based selection into a unified framework. All models adopt the same chronological data split (70% training, 10% validation, 20% testing), optimization settings, and evaluation metrics, including RMSE, MAE, $R^{2}$, and MAPE. Improvements in predictive goodness of fit are reported as $\Delta R^{2}$, calculated relative to the FALSTM baseline within each region (Table 2).

**Table 2:** Regional component contribution analysis.

Model Variant	RMSE	MAE	$R^{2}$	$\Delta R^{2}$	RMSE	MAE	$R^{2}$	$\Delta R^{2}$
	Northern China				Southern China			
FALSTM (Baseline)	0.0789	0.0567	0.6234	–	0.1187	0.0834	0.4987	–
AFD-FALSTM	0.0745	0.0512	0.6789	0.0555	0.1123	0.0789	0.5456	0.0469
SHAP-FALSTM	0.0723	0.0489	0.6923	0.0689	0.1089	0.0756	0.5623	0.0636
FaXNet (Proposed)	0.0698	0.0411	0.7239	0.1005	0.0889	0.0616	0.6778	0.1791

Across both regions, each component yields consistent and complementary gains. AFD delivers sizeable improvements over the baseline (Δ$R^{2}$ = + 0.0555 north; +0.0469 south), which is consistent with the notion that data-adaptive frequency representations can better capture multi-scale temporal structure than raw-series modeling alone. SHAP-based selection provides further benefits (Δ$R^{2}$ = + 0.0689 north; +0.0636 south), suggesting that restricting the model to a smaller set of high-attribution components may reduce redundant variability and improve generalization under the same training protocol. Notably, the combined model outperforms either single-addition variant in both regions, showing that combining AFD and SHAP-based selection yields larger gains than introducing either component alone, suggesting largely additive benefits under the current setting. The larger absolute $\Delta R^{2}$ in southern China may reflect greater benefit from adaptive decomposition and principled selection under more variable dynamics, although other factors such as regional data characteristics and unmodeled drivers may also contribute to this disparity.

### 3.3 Feature selection sensitivity analysis

To determine an appropriate feature configuration and characterize the trade-off between predictive performance and model complexity, we conducted a systematic evaluation of SHAP-selected features ranging from 4 to 16 components in both northern and southern China. This analysis quantifies how increasing feature dimensionality affects accuracy (RMSE, MAE, $R^{2}$), with model complexity characterized by the number of selected components (K) (Table 3).

**Table 3:** Regional feature selection trade-off analysis.

Features	RMSE	MAE	$R^{2}$	RMSE	MAE	$R^{2}$
	Northern China			Southern China		
4	0.0756	0.0523	0.6789	0.0923	0.0645	0.6123
6	0.0734	0.0498	0.6923	0.0901	0.0623	0.6345
8	0.0721	0.0476	0.7056	0.0892	0.0612	0.6523
10	0.0712	0.0456	0.7123	0.0887	0.0608	0.6654
12	**0.0698**	**0.0411**	**0.7239**	**0.0889**	**0.0616**	**0.6778**
14	0.0705	0.0418	0.7184	0.0895	0.0621	0.6712
16	0.0704	0.0416	0.7195	0.0898	0.0624	0.6689

Bold values indicate the best performance for each metric within the corresponding region.

**Figure 4:**
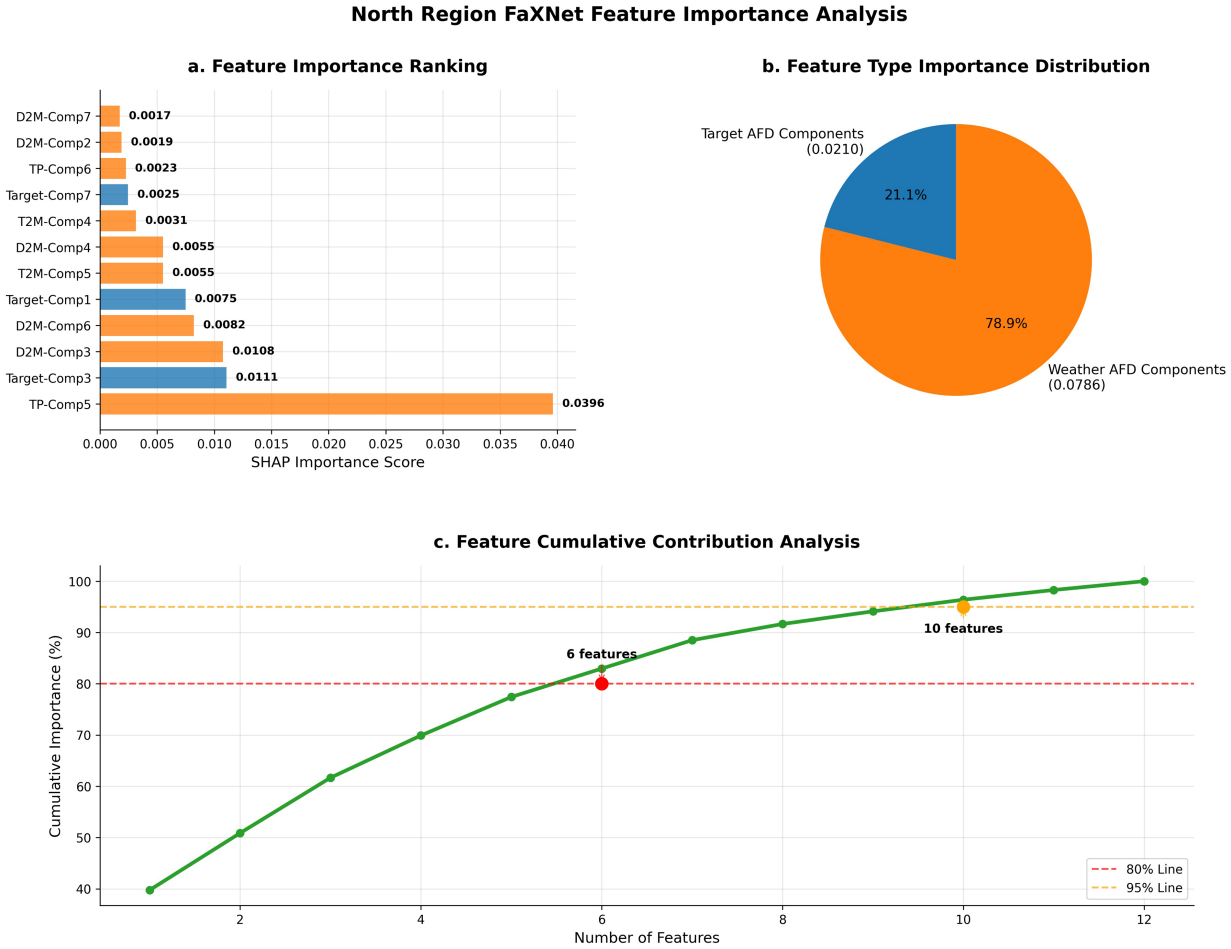
FaXNet feature importance analysis for Northern China. SHAP-based feature attribution illustrating the relative contributions of frequency components and meteorological variables. Panels show **(a)** individual feature importance ranking, **(b)** type-wise importance distribution between target and weather-derived components, and **(c)** cumulative contribution analysis identifying the optimal number of features for accurate prediction with a compact feature set.

The results indicate that selecting 12 features provides the most balanced configuration across regions, yielding the optimal trade-off between model complexity and predictive accuracy. At this configuration, performance reaches its peak in Northern China with $R^{2}\;$= 0.7239 and remains strong in Southern China with $R^{2}\;$= 0.6778, consistent with earlier regional analyses. Accuracy generally improves as K increases from 4 to 12, suggesting that moderate expansion of the selected set captures additional informative components beyond smaller subsets. When the number of features exceeds 12, accuracy begins to decline in both regions, a pattern consistent with diminishing marginal returns from additional components and increased redundancy under a fixed training protocol. These results support that SHAP-based selection can identify a compact subset associated with strong generalization under the current setting. Notably, this sensitivity analysis does not by itself establish causal “noise suppression”; rather, it provides empirical evidence that a compact SHAP-selected subset is associated with the best observed generalization under the current data and modeling assumptions (Figure 4).

### 3.4 Interpretability analysis

**Figure 5:**
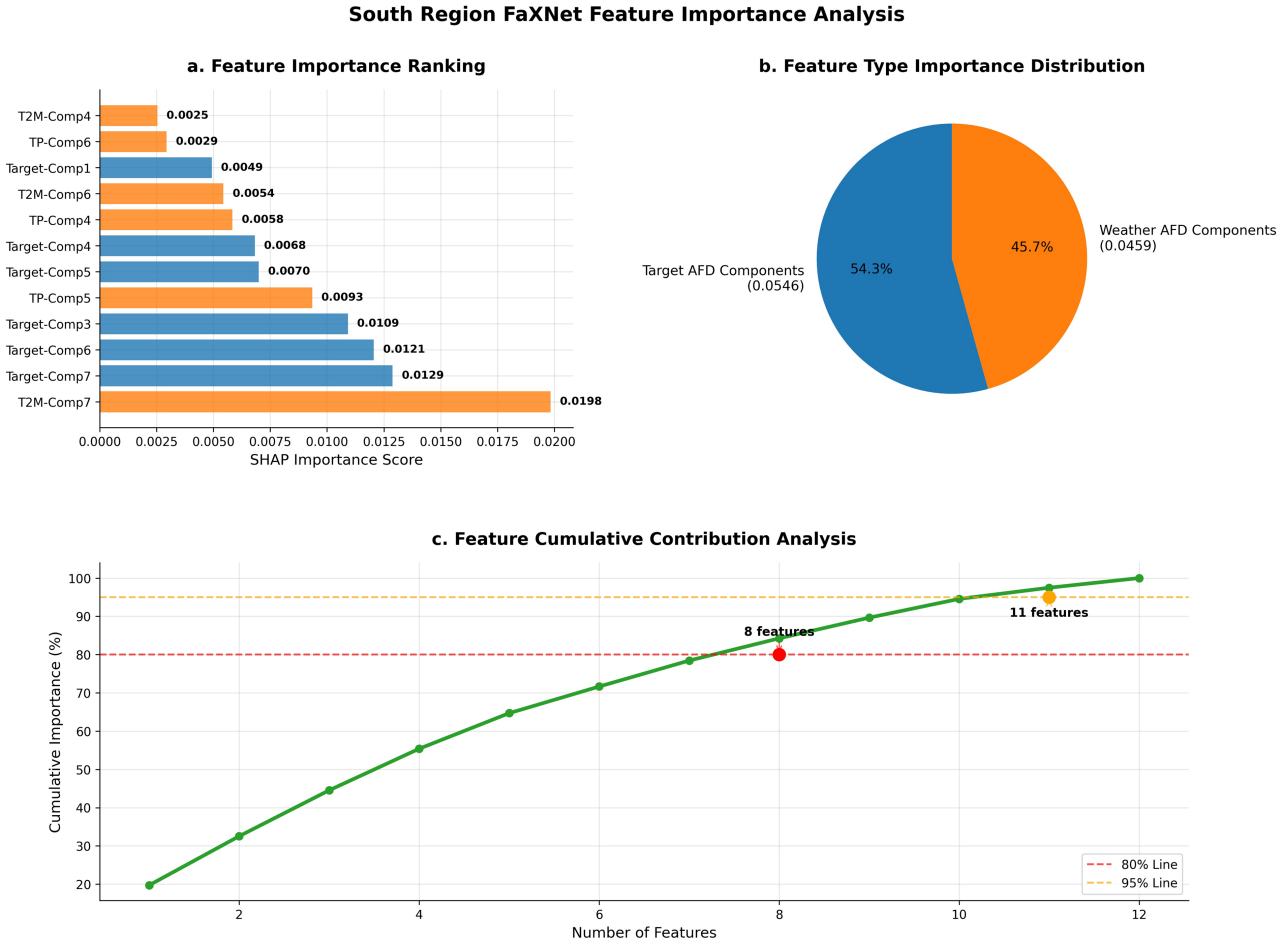
FaXNet feature importance analysis for Southern China. SHAP-based feature attribution illustrating the relative contributions of frequency components and meteorological variables. Panels show **(a)** individual feature importance ranking, **(b)** type-wise importance distribution between target and weather-derived components, and **(c)** cumulative contribution analysis identifying the optimal number of features for accurate prediction with a compact feature set.

Understanding the factors associated with influenza predictions is crucial for both model validation and public health applications. Our framework’s SHAP-based interpretability analysis provides quantitative insights into the relative importance of different meteorological and temporal features across diverse climatic regions, supporting transparent model auditing and region-specific interpretation under a consistent attribution protocol (Figure 5).

#### 3.4.1 Regional SHAP feature importance analysis

To systematically evaluate feature contributions, we employ SHAP (SHapley Additive exPlanations) analysis on the trained models for both regions. This game-theoretic approach quantifies the marginal contribution of each feature to the final prediction, providing interpretable and theoretically grounded feature importance rankings (Table 4).

**Table 4:** Top feature importance scores by region.

Rank	Component	Series	Importance	Component	Series	Importance
	Northern China			Southern China		
1	5	north_tp	0.0396	7	south_t2m	0.0198
2	3	target	0.0111	7	target	0.0129
3	3	north_d2m	0.0108	6	target	0.0121
4	6	north_d2m	0.0082	5	target	0.007
5	1	target	0.0075	4	target	0.0068

#### 3.4.2 Regional transmission mechanism analysis

The SHAP rankings highlight regional differences in the relative roles of meteorological covariates and target-derived temporal components. In northern China, precipitation (north_tp, component 5) is the most influential feature (importance = 0.0396), exceeding the next-ranked feature by a large margin. This concentration of importance is consistent with a stronger reliance on meteorological conditions for predictive signal in the temperate north, where seasonal regularity is often stronger and meteorological covariates can be more informative under the current feature set (37). Importantly, SHAP importance reflects association within the model and dataset rather than causal effect, and should therefore be interpreted as indicating which variables the model most relies upon for prediction given the available inputs.

In southern China, temperature (south_t2m, component 7) is the top-ranked meteorological driver (importance = 0.0198), while several target-derived components also appear among the highest-ranked features (e.g., target component 7: 0.0129; target component 6: 0.0121). The more distributed importance pattern indicates that the model’s predictions are informed by both meteorological covariates and endogenous temporal structure captured by the target-series components. This pattern does not by itself establish a distinct “transmission mechanism,” but is consistent with the possibility that subtropical influenza dynamics are shaped by multiple interacting factors and may be less dominated by a single meteorological proxy under the current covariate set.

#### 3.4.3 Implications for surveillance and decision support

The regional differences in feature importance have implications for influenza surveillance and intervention planning. The precipitation-dominant attribution pattern in northern China suggests that meteorology-informed monitoring may be particularly useful for contextualizing forecast changes in temperate settings. In contrast, the more distributed importance pattern in southern China suggests that incorporating additional contextual signals (e.g., mobility, school terms, vaccination uptake, or virological indicators) may further improve interpretability and robustness, especially if these drivers explain variability not captured by the current covariates.

Overall, these interpretability results support the view that FaXNet can produce region-sensitive attributions within a unified modeling pipeline, enabling stakeholders to inspect which inputs are most influential for forecasts in different climatic contexts. SHAP-based explanations enhance transparency for model auditing and communication, but should be interpreted alongside predictive performance, uncertainty estimates, and domain knowledge rather than as standalone causal evidence.

## 4 Conclusion

This study proposed FaXNet, a unified framework integrating adaptive spectral decomposition, SHAP-guided selection, and probabilistic deep learning to deliver transparent influenza forecasts. Extensive experiments demonstrated that FaXNet achieved superior accuracy and robustness across northern and southern China compared to state-of-the-art baselines. Crucially, the interpretability analysis elucidated distinct regional drivers, specifically precipitation dominance in the north versus complex thermal-social interactions in the south, providing a transparent scientific basis for risk-sensitive public health interventions.

Future research should prioritize three actionable directions to enhance operational viability. First, to enable real-time large-scale deployment, engineering accelerations such as warm-start pole selection or approximate SHAP algorithms are recommended to mitigate computational overhead. Second, to deepen causal understanding beyond meteorological correlations, future iterations should incorporate socio-behavioral covariates, including inter-regional mobility data and vaccination coverage, to better capture the complex human-driven factors shaping transmission dynamics. Finally, we propose extending this modality-agnostic framework to other multi-scale pathogens, such as dengue or RSV, to rigorously test its generalizability, ultimately advancing the paradigm of explainable and uncertainty-aware precision epidemiology.

## Data Availability

The original contributions presented in the study are included in the article/supplementary material, further inquiries can be directed to the corresponding author.

## Glossary

AFD: Adaptive Fourier Decomposition
ARIMA: Autoregressive Integrated Moving Average
AdamW: Adaptive Moment Estimation with decoupled weight decay
CNIC: Chinese National Influenza Center
CRPS: Continuous Ranked Probability Score
d2m: 2-m Dew-Point Temperature
ERA5-Land: ECMWF Reanalysis v5-Land
FaXNet: Frequency-Adaptive eXplainable Network
FALSTM: Frequency-Adaptive Long Short-Term Memory
FFT: Fast Fourier Transform
GELU: Gaussian Error Linear Unit
GRU: Gated Recurrent Unit
LN: Layer Normalization
LSTM: Long Short-Term Memory
MAE: Mean Absolute Error
MAPE: Mean Absolute Percentage Error
MLP: Multilayer Perceptron
MSE: Mean Squared Error
NLL: Negative Log-Likelihood
PIT: Probability Integral Transform
$R^{2}$: Coefficient of Determination
ReLU: Rectified Linear Unit
RMSE: Root Mean Squared Error
SHAP: SHapley Additive exPlanations
SVR-RBF: Support Vector Regression with Radial Basis Function kernel
t2m: 2-m Air Temperature
tp: Total Precipitation
EMD: Empirical Mode Decomposition
VMD: Variational Mode Decomposition
XAI: Explainable Artificial Intelligence
