# Correction: FaXNet: a frequency-adaptive, explainable, and uncertainty-aware network for influenza forecasting

**DOI:** 10.3389/fpubh.2026.1804925

**Published:** 2026-02-24

**Authors:** Wei He, Xuanfeng Li, Xiaolin Liang, Zige Liu, Zhiqi Zeng, Zifeng Yang, Chitin Hon

**Affiliations:** 1 Faculty of Innovation Engineering, Macau University of Science and Technology, Macao, Macao SAR, China; 2 Institute of Systems Engineering, Macau University of Science and Technology, Macao, Macao SAR, China; 3 Faculty of Social Sciences, The University of Hong Kong, Hong Kong, Hong Kong SAR, China; 4 National Clinical Research Center for Respiratory Disease, State Key Laboratory of Respiratory Disease, Guangzhou Institute of Respiratory Health, The First affiliated Hospital of Guangzhou Medical University, Guangzhou, Guangdong, China

**Keywords:** Adaptive Fourier Decomposition (AFD), explainable AI (XAI), frequency-adaptive modeling, influenza forecasting, Long Short-Term Memory (LSTM), SHAP (SHapley Additive exPlanations)

In the published article, there was an error in the ordering of the affiliations.

Affiliation 3 was erroneously listed as “National Clinical Research Center for Respiratory Disease, State Key Laboratory of Respiratory Disease, Guangzhou Institute of Respiratory Health, The First affiliated Hospital of Guangzhou Medical University, Guangzhou, Guangdong, China”. It is corrected to: “Faculty of Social Sciences, The University of Hong Kong, Hong Kong, Hong Kong SAR, China”.

Affiliation 4 was erroneously listed as “Faculty of Social Sciences, The University of Hong Kong, Hong Kong, Hong Kong SAR, China”. It is corrected to: “National Clinical Research Center for Respiratory Disease, State Key Laboratory of Respiratory Disease, Guangzhou Institute of Respiratory Health, The First affiliated Hospital of Guangzhou Medical University, Guangzhou, Guangdong, China”.

The authors' superscripts match these corrected affiliations.

The original version of this article has been updated.

